# Supplementary material for: The Association of Socioeconomic Status, the Concern for Catching Covid-19, and Anxiety Between Individuals with and without a Cancer History from a Cross-sectional Study
Source: medRxiv. 2022 Jul 29:2022.07.26.22278080. Preprint. [Version 1] doi: 10.1101/2022.07.26.22278080 (PMC9347286; doi:10.1101/2022.07.26.22278080)
Supplement: 1 [file NIHPP2022.07.26.22278080v1-supplement-1.pdf]

Supplementary Table 1. Demographic characteristics for included and excluded participants

Variable		All responded participants (n=9280)	Participants included (n=7012)	Participants excluded (n=2268)	P-value
Age					
	≤40 yrs	1349 (14.54%)	997 (14.22%)	352 (15.52%)	<.0001
	41-60 yrs	3786 (40.8%)	3012 (42.95%)	774 (34.13%)	
	≥61 yrs	4074 (43.9%)	3003 (42.83%)	1071 (47.22%)	
	Missing	71 (0.77%)	0 (0%)	71 (3.13%)	
Sex					
	Male	3102 (33.43%)	2260 (32.23%)	842 (37.13%)	<.0001
	Female	6130 (66.06%)	4752 (67.77%)	1378 (60.76%)	
	Missing	48 (0.52%)	0 (0%)	48 (2.12%)	
Race					
	White	8201 (88.37%)	6279 (89.55%)	1922 (84.74%)	0.1317
	Black	559 (6.02%)	412 (5.88%)	147 (6.48%)	
	Asian	162 (1.75%)	122 (1.74%)	40 (1.76%)	
	Other/Multiple	245 (2.64%)	199 (2.84%)	46 (2.03%)	
	Missing	113 (1.22%)	0 (0%)	113 (4.98%)	
SES score					
	Low (0-5)	1754 (18.9%)	1601 (22.83%)	153 (6.75%)	<.0001
	Intermediate (6-8)	3081 (33.2%)	2929 (41.77%)	152 (6.7%)	
	High (9-10)	2569 (27.68%)	2482 (35.4%)	87 (3.84%)	
	Missing	1876 (20.22%)	0 (0%)	1876 (82.72%)	
